# Canine vector-borne diseases: a changing world demands a new preventive strategy from veterinarians

**DOI:** 10.1186/s13071-026-07331-2

**Published:** 2026-03-06

**Authors:** Filipe Dantas-Torres, Domenico Otranto

**Affiliations:** 1 Department of Immunoparasitology, Aggeu Magalhães Institute, , Fundação Oswaldo Cruz (Fiocruz), Recife, Brazil; 2 Department of Veterinary Medicine, University of Bari, Valenzano, Italy; 3 Department of Veterinary Clinical Sciences, City University of Hong Kong, Hong Kong, China

**Keywords:** Ticks, Sand flies, Mosquitoes, Fleas, Dogs, Zoonosis, Prevention, Pet owners, Veterinarians

## Abstract

Canine vector-borne diseases pose a continuous threat to dogs worldwide. Various vector-borne agents, including bacteria, protozoa, helminths, and, to a lesser extent, viruses, cause these diseases. These pathogens are transmitted primarily by arthropod vectors, including ticks, mosquitoes, phlebotomine sand flies, fleas, lice, tabanid flies and triatomine bugs. The diagnosis and treatment of these diseases can be challenging, and co-infections may further complicate management. Besides being potentially fatal to dogs, some of these pathogens are zoonotic. Many biotic and abiotic factors (e.g. climate change, increased mobility of people and animals, urban expansion and land-use changes) are affecting the distribution and activity of vectors and the pathogens they transmit worldwide. The evolving relationship between dogs and humans may also increase owners’ exposure to ectoparasites and the diseases they transmit. In this article, we review key aspects of canine vector-borne diseases and discuss the importance of year-round prevention, as recommended by established international guidelines from leading veterinary parasitology organizations.

**Graphical Abstract:**

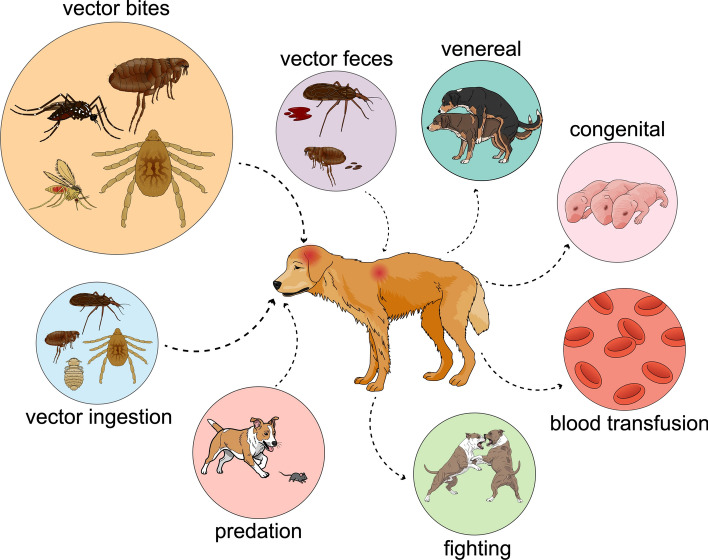

## Background

**Fig. 1:**
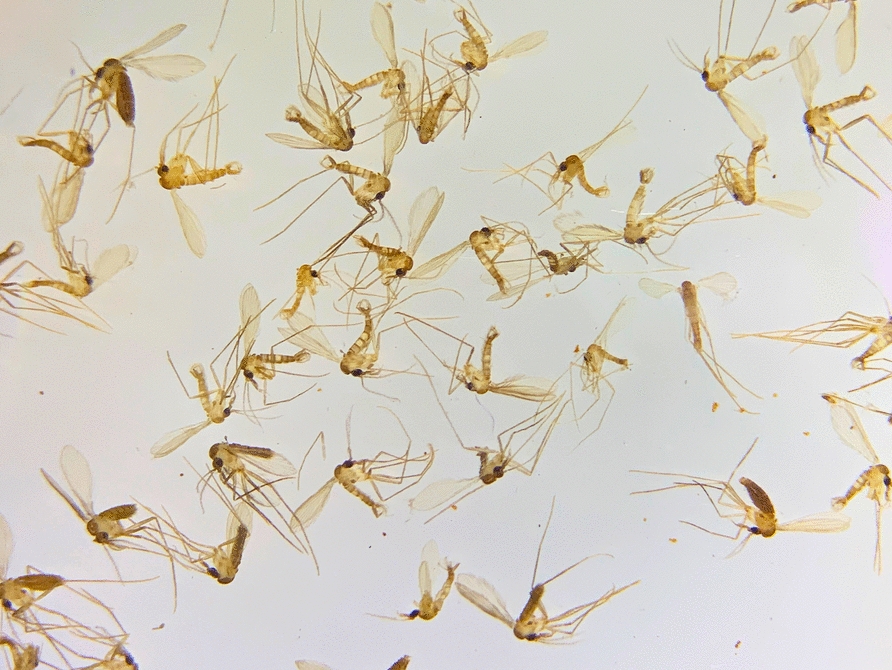
Sand flies of the species *Lutzomyia longipalpis* are tiny and fragile insects, yet they hold immense significance for public health

Vector-borne diseases pose a significant threat to dogs and humans worldwide. Their prevalence is exceptionally high in tropical and subtropical regions [1, 2], but they are also prevalent in temperate areas, where global warming is making conditions favourable for vector establishment in places where they were previously absent. A categorical example is leishmaniasis caused by *Leishmania infantum*, which is expanding northwards in Europe [3, 4] and southwards in South America [5–7]. A specific example of the latter is Uruguay, which was traditionally considered one of the few countries in the Americas free from animal and human leishmaniasis for many years [8]. Following the first record of *Lutzomyia longipalpis* (Fig. 1), the primary vector of *L. infantum* in the Americas [9], an outbreak of canine leishmaniasis was detected in the country [5]. Since then, cases of human visceral leishmaniasis caused by *L. infantum* have also been reported [6], illustrating the recent expansion of a significant vector-borne disease after the introduction of its vector.

Another critical group of vector-borne diseases is the tick-borne diseases, which include canine monocytic ehrlichiosis, caused by *Ehrlichia canis*, and babesiosis, caused by *Babesia vogeli*. These diseases are highly prevalent in the tropics and subtropics, where tick vectors are present year-round and complete several generations per year [10]. Their epidemiology is complex, involving multiple vectors in diverse settings across regions. Brown dog ticks (*Rhipicephalus sanguineus* sensu lato) are commonly involved in the transmission of these pathogens [11]. In tropical regions, *E. canis* and *B. vogeli* are mainly transmitted by *Rhipicephalus linnaei* [11]. In contrast, *Rhipicephalus sanguineus* or other related species (e.g. *Rhipicephalus rutilus*) are the probable vectors in temperate regions [11, 12]. Still, other ticks, such as *Amblyomma aureolatum*, can also transmit clinically relevant pathogens to dogs, including *Babesia vitalii* (also known as *Rangelia vitalii*), the causative agent of rangeliosis in southeastern and southern Brazil, Uruguay and Argentina [13]. Incidentally, this tick is also a vector of *Rickettsia rickettsii*, the agent of spotted fever rickettsiosis, in some areas of southeastern Brazil [14]. In other foci in Brazil, *R. rickettsii* is transmitted by *Amblyomma sculptum*, and capybaras serve as amplifying hosts [15]. In the southwestern border area of the USA and across northern Mexico, brown dog ticks are the primary vectors of *R. rickettsii* [16]. In contrast, *Dermacentor variabilis* and *Dermacentor andersoni* are the primary vectors in other areas of the USA [17]. These examples exemplify the complexity of a tick-borne disease, which can involve multiple vectors in diverse epidemiological scenarios.

While factors underlying changes in vector and pathogen distributions are sometimes poorly understood, current understanding is that climate and environmental changes play a significant role, as do the movements of animal and human populations [18–20]. The changing distribution of pathogens and vectors, along with the dynamic epidemiological scenarios of the diseases they cause, necessitate ongoing updates and the implementation of new approaches to prevent these diseases, particularly those of zoonotic concern.

 In this article, we review key aspects of canine vector-borne diseases and discuss the importance of year-round prevention. This is a narrative review informed by extensive field experience rather than a systematic review. Our literature search included key references, recent publications retrieved in PubMed and guidelines from international animal parasite councils.

## Transmission pathways: mainly vector bites

Vector-borne pathogens are primarily transmitted to dogs by blood-feeding arthropod vectors, a key consideration when choosing prevention strategies. The transmission times and mechanisms by which pathogens reach the host may vary with the feeding habits of the vectors and the biology of the pathogens they transmit [21–24]. For example, third-stage larvae of *Dirofilaria immitis*, the canine heartworm, migrate actively from the female mosquito’s mouthparts to the host’s skin, deposited in a drop of haemolymph, penetrating through the puncture site immediately after a blood meal [25]. The transmission of *L. infantum* occurs when the female sand fly regurgitates the promastigote secretory gel that blocks the stomodaeal valve before it can blood-feed [26]. During this process, both the gel and metacyclic promastigotes are deposited into the host’s skin, promoting transmission [26].

Mosquitoes and sand flies are short blood feeders, and the transmission of both *D. immitis* and *L. infantum* occurs within minutes. In contrast, tick-borne pathogens may require minutes to days to be transmitted [22, 23]. While the transmission of tick-borne viruses can occur within 15–60 min [23], bacteria and protozoa are inoculated into the host over a variable period, ranging from approximately 3 h for *Ehrlichia canis* [27] to 24–48 h for *Borrelia* spp. and *Babesia* spp. [23]. However, specific events in nature (e.g. interrupted feeding) may affect transmission dynamics. For example, interrupted feeding can shorten the transmission time, as demonstrated for *R. rickettsii* transmission by *A. aureolatum* [23]. Under experimental conditions, unfed nymphs and adults were found to require at least 10 h to transmit *R. rickettsii*, but fed ticks needed only 10 min [28]. While it is unlikely that a fed tick will feed on a human, this may happen with dogs infested with multiple ticks. In either case, ticks should be removed as soon as they start feeding to reduce the risk of pathogen transmission [17].

Another consideration is that not all developmental stages of arthropod vectors are capable of transmitting pathogens to dogs. For instance, female mosquitoes feed on both nectar and blood, whereas males feed exclusively on nectar from various sources [29]. The same holds for sand flies [30]. Similarly, most pathogens, except viruses, are not transmitted transovarially by mosquitoes and sand flies [31, 32], implying that females must first acquire the infection from an infected host and subsequently transmit it to an uninfected host. Yet all developmental stages of veterinary-significant hard ticks feed on blood.

Additionally, transstadial and transovarial transmission are standard for many tick-borne pathogens [11]. This means that the risk of tick-borne pathogen transmission in some areas may be higher than that of mosquito- and sand fly-borne pathogens, such as *D. immitis* and *L. infantum*, respectively. Likewise, cat fleas can pass *Rickettsia felis* to their offspring via transovarial transmission, whereas this mode of transmission does not seem to occur with *Bartonella henselae* [33, 34].

**Fig. 2:**
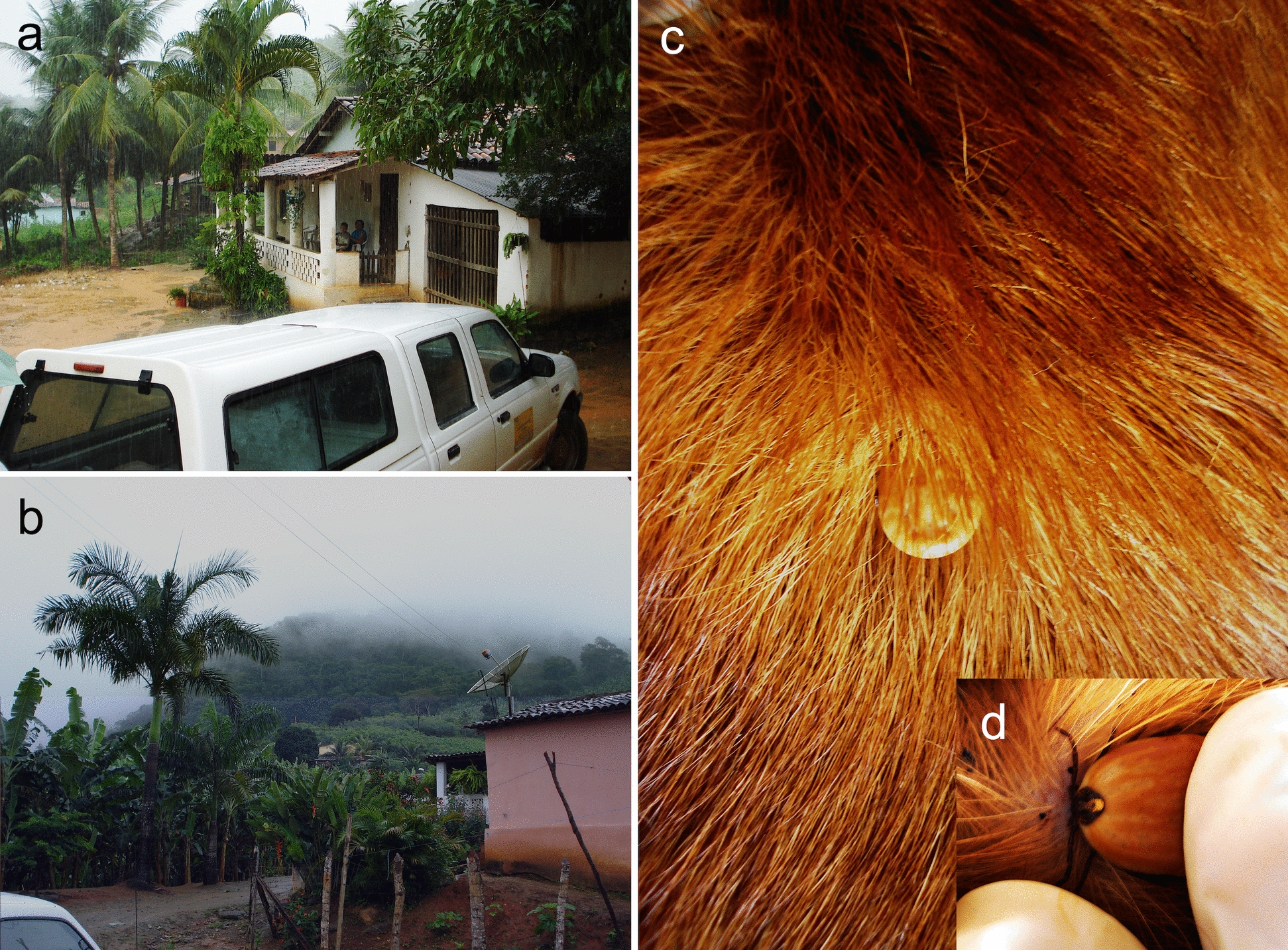
Remote rural areas (**a**, **b**) in northeastern Brazil, where *Hepatozoon canis* is enzootic and transmitted by *Amblyomma ovale* (**c**). **d** Close-up view of the dorsal scutum of the engorging female shown in **c**

While vector bite is the primary route of transmission for vector-borne pathogens, there are exceptions. For example, *Hepatozoon canis* and *Hepatozoon americanum* are transmitted when dogs ingest infected ticks that contain mature oocysts (Fig. 2) [35]. For *H. americanum*, predation has also been shown to be a possible route of transmission [36].

Another exception is *Trypanosoma cruzi*, which is primarily transmitted when an infected triatomine bug defecates on the dog’s skin during blood-feeding [37]. Nonetheless, the risk of oral transmission through the ingestion of infected triatomine bugs is also high [38, 39]. Transmission via contamination of skin lesions with flea faeces is likely the primary route by which *B. henselae* spreads among mammals [34, 40]. Bite is also considered an essential route of transmission for *Babesia gibsoni* among fighting dogs [41]. Finally, some vector-borne pathogens can be transmitted to dogs through blood transfusion, sexual contact or congenitally [42–44]. All of these possible transmission routes should be considered when assessing the risk of vector-borne pathogen transmission in dogs, including in geographical areas where the primary vectors are absent. This will ultimately help veterinary practitioners establish individual recommendations (e.g. avoiding reproduction, exclusion from blood donation programmes) in addition to vector control.

## Vector-borne diseases pose a year-round risk, but the risk for specific diseases may vary regionally

The primary risk factor for vector-borne pathogen infections is exposure to their vectors. In tropical and subtropical regions, these vectors are present year-round because the favourable climate supports their year-round survival. This is the typical case of brown dog ticks, which are present year-round in most areas where they occur [45, 46]. Even in temperate regions, climate change is extending the transmission seasons, eventually altering the distribution of certain vector-borne diseases, including canine leishmaniasis (see below).

An example of this are babesiosis and ehrlichiosis, caused by pathogens transmitted by brown dog ticks, including *R. linnaei* and *R. sanguineus* [11]. Dogs living in areas where *R. linnaei* is present are at a higher risk of ehrlichiosis compared to those living in areas where *R. sanguineus* is present when it is taken into consideration that the former is a more competent vector [47]. While the prevalence of *E. canis* may be higher in tropical regions [2], this bacterium is also present in temperate areas [12]. This is also true for *B. vogeli*, which is highly prevalent in tropical regions [2] but is also present in temperate regions [48–52]. The risk of tick infestations and tick-borne infections may also vary between urban and rural areas. In Brazil, for example, brown dog ticks are more prevalent among dogs living in urban or suburban areas than in rural areas [53, 54]. In rural areas, dogs are thought to be less exposed to brown dog ticks than to *Amblyomma* spp., including *Amblyomma ovale* and *A. aureolatum* [54–56]. A study conducted in southeastern Brazil found that *A. aureolatum* was more prevalent at higher altitudes, while *A. ovale* was more prevalent at lower altitudes [55]. Considering that *A. ovale* and *A. aureolatum* may transmit *H. canis* and *B. vitalii*, respectively, the risk of infection by these pathogens may vary altitudinally. Therefore, *B. vitalii* appears to be restricted to southeastern and southern Brazil, Argentina and Uruguay, and *A. aureolatum* is the primary vector [57]. Yet *H. canis* is widespread across the Americas [1], and other vectors are suspected to be involved, including brown dog ticks and *Haemaphysalis longicornis* in the USA [58].

While *Amblyomma* ticks may prevail in some rural areas, this is not always the case. In some rural areas of Brazil, brown dog ticks may be more prevalent, with dogs being highly exposed to *E. canis* [59]. A similar situation has been documented on indigenous reservations in western Arizona, where brown dog ticks not only prevailed but were also confirmed to play a primary role in *R. rickettsii* transmission [60, 61]. Again, this shows that dogs living in different environments may be exposed to different ticks and associated pathogens.

Another critical example is heartworm disease caused by *D. immitis*. There appears to be a regional variation in the risk of heartworm infection in dogs [62]. In the USA, where millions of dogs are screened annually for heartworm antigens, data indicate that county-level prevalence varies across the country, with higher prevalence along most of the Atlantic coast, in the central USA and in the western states [63]. Clusters of decreasing prevalence were reported along the Mississippi Alluvial Plain, Oklahoma, Kansas and Florida [63]. Predictive models indicate favourable temperatures for heartworm transmission in most South American countries, with seasonal transmission in some areas (e.g. Argentina, Chile, Uruguay, eastern Paraguay and southeastern Brazil) [64]. Increasing temperatures could extend the transmission season in some areas, a particular concern in regions where adulticide treatment is unavailable [65]. In some endemic countries, dogs living in coastal areas are at a significantly higher risk than those in the countryside, with heartworms rare or absent in certain regions [2, 66, 67]. Similarly, the southernmost focus of heartworm in Europe was identified on the small island of Linosa (Sicily), where 58.9% of tested dogs tested positive [68]. Meanwhile, *Dirofilaria* spp. are expanding in Eastern European countries, with an increasing number of human cases recorded over the past 20 years [69]. Again, this illustrates regional and seasonal variations in the risk of vector-borne disease transmission, which are directly influenced by the environments and climates in which dogs reside, as well as the vectors and pathogens associated with them.

Canine leishmaniasis is another paradigmatic example of such variation in distribution patterns. This disease is caused by a range of *Leishmania* species in the Americas, from the USA to Argentina and Uruguay [7]. It has long been considered absent in Chile, which has an extraordinary terroir for wine production, but a mostly unsuitable climate for phlebotomine sand flies. Recent studies have reported the presence of *Leishmania* DNA or anti-*Leishmania* antibodies in dogs from Chile [70, 71]. However, the *Leishmania* species was not identified, and the autochthonous nature of these few infections remains to be proven. While widespread across the Americas, regional variations in leishmaniasis transmission risk do exist. Canine leishmaniasis is more prevalent in tropical than in temperate areas, likely due to the higher abundance of phlebotomine sand flies in the former. The risk of infection by different *Leishmania* species may vary, as dogs from rural and forested areas are often exposed to native parasites, such as *Leishmania braziliensis*, *Leishmania panamensis* and *Leishmania mexicana* [7]. These species exhibit a primary sylvatic life cycle, with forest rodents serving as the primary reservoirs and forest-dwelling phlebotomine sand flies as the primary vectors [72]. Nonetheless, deforestation and the construction of rural properties near forests may favour the domestication of the transmission cycle of these parasites, as is the case with *L. braziliensis* and *L. panamensis* [73, 74].

**Fig. 3:**
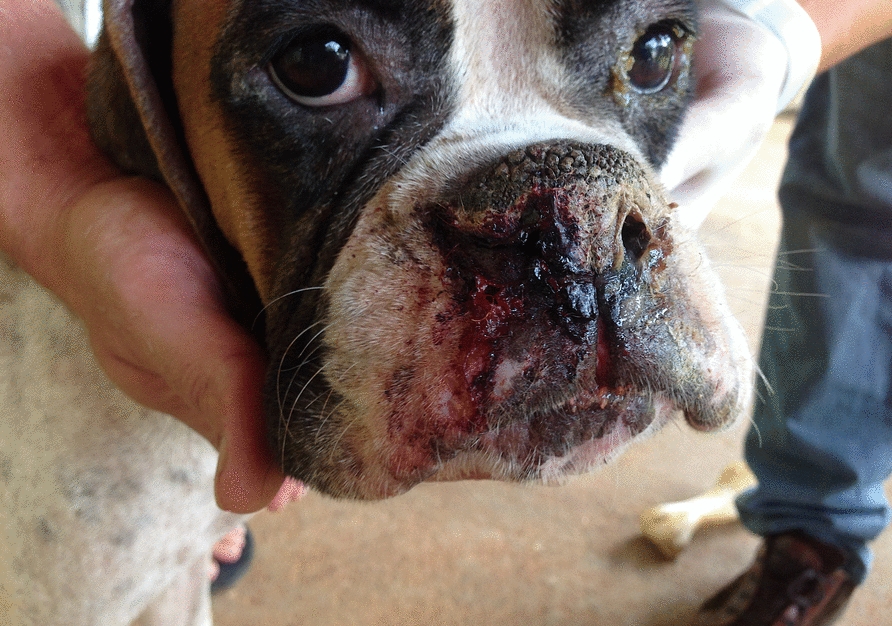
A recurrent case of leishmaniasis in a dog owned by a person living in an urban area in Brasilia, Brazil. The dog presented bilateral periocular alopecia, bleeding from the nose and nose hyperkeratosis, along with generalized dermatitis and other clinical signs

In contrast, *L. infantum* is primarily associated with rural and urban dogs (Fig. 3), which serve as primary reservoirs of this parasite [7]. The primary vector (*Lutzomyia longipalpis*) is widespread across the Americas, from Uruguay to Mexico [75]. Furthermore, other vectors are involved in specific regions [76], indicating that a significant number of dogs, cats and humans across the Americas are at risk of infection with *L. infantum*.

The above examples demonstrate that the risk of vector-borne infections can vary locally, primarily due to factors affecting the vectors, including landscape and climate. Nonetheless, they also indicate that dogs living in rural and urban areas, in both tropical and temperate regions, are exposed to a wide range of vector-borne pathogens, which are primarily transmitted year-round.

## Prevention of vector-borne infections in dogs: the international guidelines

The Companion Animal Parasite Council (CAPC; https://capcvet.org), the European Scientific Council Companion Animal Parasites (ESCCAP; https://www.esccap.org) and the Tropical Council for Companion Animal Parasites (TroCCAP; https://www.troccap.com) have elaborated guidelines for the prevention of vector-borne infections in dogs and cats. The American Heartworm Society (AHS) also provides guidelines for heartworm prevention in dogs and cats (https://www.heartwormsociety.org).

There is consensus that veterinarians in tropical and subtropical regions should recommend year-round prevention [77], as dogs in these regions face year-round infection risk. Even in temperate areas, dogs are exposed to various vector-borne infections, some of which are zoonotic. For example, canine leishmaniasis, once a typical tropical disease, is expanding into temperate regions where it was previously absent. This means that veterinarians working in temperate regions should also consider year-round prevention based on local and individual risk assessments. The use of an approved veterinary product and adherence to label recommendations should be emphasized to ensure safety and reduce the risk of drug resistance.

The AHS recommends year-round prevention for canine heartworm (https://www.heartwormsociety.org/preventives). The ESCCAP recommends preventing infection with *L. infantum* during the sand fly season (typically from April to November); however, their guidelines emphasize that year-round prevention may be advisable in some regions, including southern Spain, Italy, Portugal and Greece (https://www.esccap.org/uploads/docs/32ir16g1_0775_ESCCAP_Guideline_GL5_20241203_1p.pdf). In this context, the 4% deltamethrin collars are registered for 12 months of protection against sand flies in these countries. Consequently, even if winter temperatures may inhibit sand fly activity for some months in these countries, the protection will last for 12 months. The same may apply to tick and flea control, as a single treatment with fluralaner injectable suspension proved to be efficacious against repeated infestations with *R. sanguineus* sensu lato and *Ctenocephalides felis* in dogs for 12 months [78–80].

The TroCCAP recommends that all pets be maintained on year-round ectoparasite control (https://www.troccap.com/wp-content/uploads/2022/01/TroCCAP-Canine-Feline-Ecto-Guidelines-English-v1.pdf), and the ESCCAP recommends year-round prevention in areas with a high risk of tick-borne pathogen transmission (https://www.esccap.org/uploads/docs/cgqtqpf1_0720_ESCCAP_GL3__English_v19_1p.pdf). Tick-borne pathogens are prevalent in southern Europe and are expanding northward, driven by rising temperatures and animal host movements that shift the distribution of tick vectors, including *Dermacentor marginatus* and *Ixodes ricinus* [18, 81]. A study in Germany found a significant association between *Babesia* spp. infection and the absence of ectoparasite prophylaxis [82].

Year-round prevention is advisable not only to protect dogs but also to protect people from zoonotic diseases. This is particularly important in areas where diseases such as visceral leishmaniasis, caused by *L. infantum*, and spotted fever rickettsiosis, caused by *R. rickettsii*, are endemic, given dogs’ role as reservoirs of *L. infantum* and as potential sources of *R. rickettsii*-infected ticks. Prevention strategies to reduce the risk of canine vector-borne diseases have been reviewed elsewhere [83], including the use of repellents and fast-killing parasiticides as first-line measures. In particular, while pyrethroids (e.g. deltamethrin, flumethrin, and permethrin) have repellent effects, isoxazolines (e.g. afoxolaner, fluralaner, lotilaner, and sarolaner) provide a systemic killing effect, which may be long-lasting (e.g. fluralaner) [83].

Although vaccines may be available to prevent canine vector-borne diseases in some countries [83], vaccines developed to date are registered to reduce the risk of disease development and do not prevent infection, underscoring the need for vector control.

## Challenges and concerns regarding year-round prevention

Determining whether a dog is at risk and, particularly, classifying this risk as low, moderate, or high, can be a challenging endeavour for the veterinary practitioner. For example, in a busy day, with a long queue of patients in the waiting room, the veterinary practitioner may often not have time for asking many questions to assess the risk level for individual patients and may, for convenience, recommend standard measures such as vaccination and deworming, often neglecting vector-borne pathogen prevention, even for dogs at high risk. Unless lice, fleas or ticks are visible on a puppy, very few veterinarians will recommend a preventative against these ectoparasites at the first consultation. This may be a significant mistake, exposing the patient to the risk of life-threatening illnesses, including babesiosis, monocytic ehrlichiosis, heartworm disease and leishmaniasis.

During the veterinary consultation, a pet owner may argue that their pet is not at risk of vector-borne infections because the pet lives in a ‘clean’ environment and has never been exposed to ticks or fleas. This argument may lead the veterinary practitioner to ponder whether the dog requires a preventive. While this is a genuine question, the veterinarian should still investigate other potential risk factors or even existing infestations that a pet owner may overlook. For example, a UK study found that half of dog and cat owners were unaware that their pets had a flea infestation, suggesting that many cases may go undetected [84]. Similarly, an indoor dog may frequently visit a pet shop for bathing and grooming, thereby being exposed to an environment infested with ticks, fleas or both. While this may seem paradoxical, many pet shops are affiliated with a veterinary facility (e.g. a clinic or hospital) where tick-infested dogs may be frequent visitors [53]. The first reported case of human infestation by *R. linnaei* in Brazil was in a house where a well-cared-for female poodle had brought back ticks from the pet shop it frequented every week for bathing and grooming [85]. The pet owner reported finding a tick attached to her arm, and ticks were subsequently found on the sofa where she had been sitting with her dog [85].

**Fig. 4:**
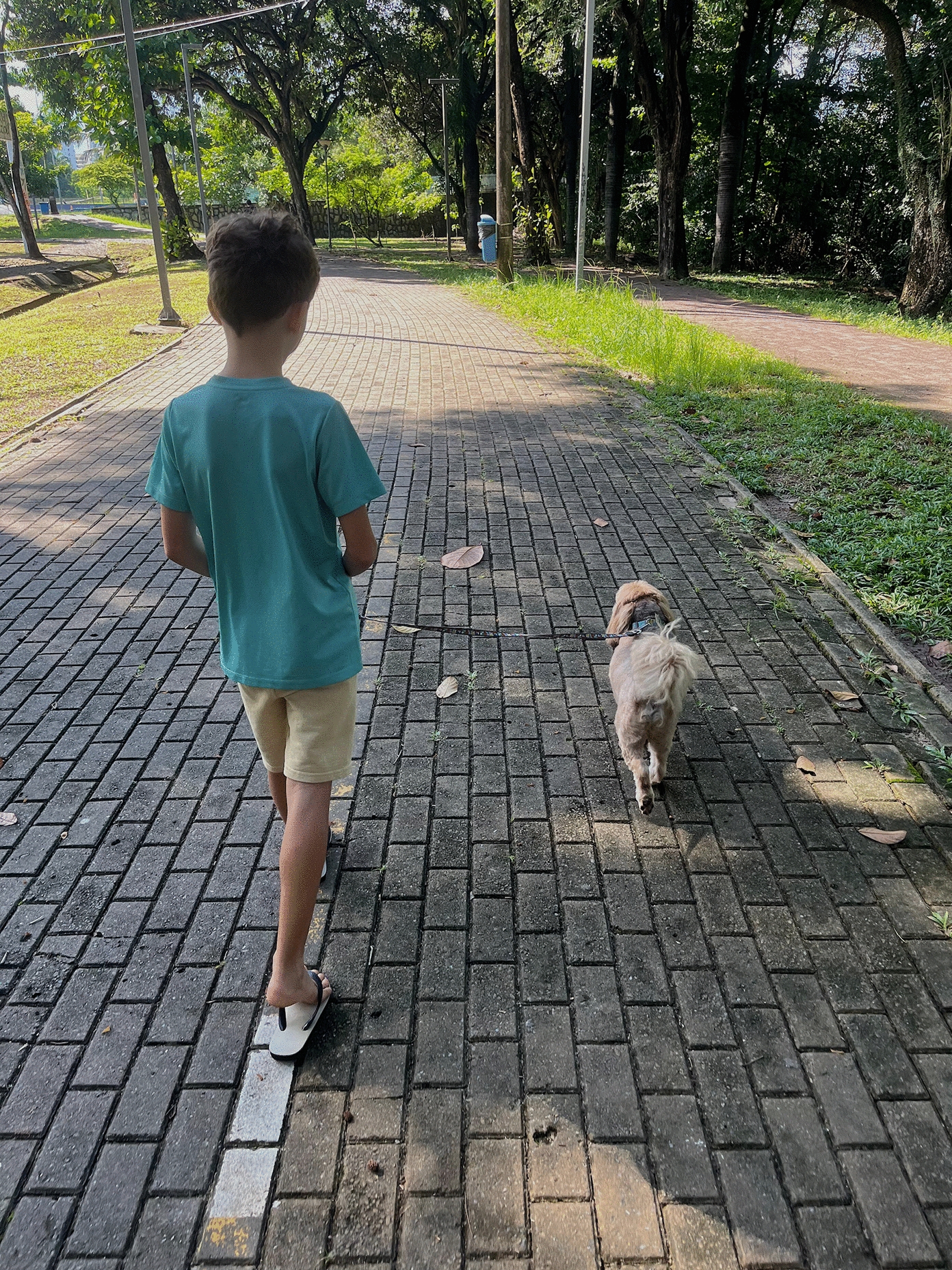
A child walking with his grandmother’s dog in a park daily visited by both stray and owned dogs. While this dog lives in an apartment, it is exposed to ticks during these walks and during his weekly visits to the pet shop for bathing

Similarly, dogs living in apartments often go for daily walks with their owners. They frequently visit parks (Fig. 4) that are possibly visited by other dogs, including strays, as well as wild animals (e.g. capybaras), and the latter may serve as blood sources for ticks, possibly contributing to local tick populations [86]. In areas where spotted fever caused by *R. rickettsii* is endemic, such walks may pose a threat to both dogs and their owners. Therefore, even if the pet owner reports an apparent low risk of vector-borne infections, the veterinary practitioner needs to critically assess all potential hidden risk factors to develop a more effective prevention strategy for each dog.

One of the primary challenges of year-round prevention is poor adherence to recommended measures [87]. Noncompliance is often due to the owner’s lack of reliable information about the clinical and zoonotic importance of vector-borne diseases and the need for prevention. It is the veterinarian’s responsibility to provide owners with accurate, evidence-based information and recommendations. In some socioeconomic contexts, however, this lack of compliance may stem from insufficient funds to afford year-round prevention, making its implementation challenging [88]. In this perspective, preventive strategies should ideally be tailored to local epidemiology, owner compliance and available resources. Public–private partnerships can still assist disadvantaged dog owners, as demonstrated in Brazil for canine leishmaniasis through the distribution of 4% deltamethrin collars via the national visceral leishmaniasis control programme [89]. This measure has been shown to be effective in reducing infection in dogs and, indirectly, in humans [90].

Additionally, noncompliance may be related to the short duration of some products and the requirement for monthly applications, which some dog owners may consider time-consuming. In this regard, prescribing long-lasting products (e.g. a single annual treatment with fluralaner injectable suspension) [80] can improve compliance. According to a US study, fluralaner’s longer re-administration interval provided dog owners with the greatest proportion of ectoparasite protection time compared with monthly treatments, which required more frequent re-dosing [91]. Indeed, long-lasting products are beneficial for pet owners who may forget to apply preventives monthly. Utilizing customer relationship management (CRM) systems, calendars or reminder applications can also help ensure preventive measures are reapplied at the recommended intervals.

Concerns regarding the overuse of parasiticides include environmental toxicity, impacts on non-target animals and the potential for resistance to these chemicals. For example, a recent UK study [92] found that spot-on products containing fipronil and imidacloprid are a significant source of surface-water pollution. The study identified pet bathing, bed washing and owner handwashing as likely sources of these chemicals [92]. The British Veterinary Association (BVA), the British Small Animal Veterinary Association (BSAVA) and the British Veterinary Zoological Society (BVZS) advise that “where reasonable and possible, products that are administered topically or through a collar should not be selected if pets swim, are having hydrotherapy or are bathed” [93]. In this regard, further research on the environmental impact of pet parasiticides is urgently needed, as recently emphasized elsewhere [94].

The growing concern over ectoparasite control in dogs [94–96] is closely linked to ongoing discussions about tick control in cattle [97]. From a vector population perspective, the pressure exerted by parasiticides applied to pet dogs is theoretically lower compared to that, for example, used to control cattle ticks. This is due not only to the significantly greater amount of chemicals used globally to control cattle ticks than those used for dogs, but also to the large refugia of dog tick populations, which are virtually never exposed to acaricides and are maintained mainly by untreated stray dogs. In confined settings (e.g. a dog shelter), failure in tick control may result in incomplete tick elimination, thereby increasing the risk of acaricidal resistance [98]. In cattle, ticks are typically controlled, rather than eliminated, to maintain enzootic stability [99], which may, in the long term, favour the selection of resistant strains. This is especially the case when products are not applied correctly (e.g. due to suboptimal dosages resulting from application errors) and when the same active ingredient is used for an extended period in a given region. Overall, the objectives and strategies for controlling cattle ticks differ significantly from those for dog ticks. Indeed, while the primary purpose of cattle tick control is to reduce tick burden and maintain enzootic stability, tick control in dogs aims to prevent contact with ticks and eliminate existing infestations on the animals, thereby reducing environmental infestation levels. Whether for cattle or dogs, the responsible use of parasiticides on animals and in the environment, in strict adherence to label instructions and international guidelines, is essential. New methods for detecting drug resistance are needed to facilitate the discovery and management of vector populations resistant to commonly used parasiticides for dogs.

Another common concern regarding the continuous use of preventive measures is safety. Often, this concern arises from misinformation or a lack of awareness regarding the safety studies conducted for each product before it is approved for the market. Veterinarians and, primarily, dog owners may be concerned about the potential effects of repeated chemical applications on their pets’ overall health. Nonetheless, there is no evidence linking the constant use of parasiticides with reduced life expectancy, chronic renal or hepatic disease. The European Medicines Agency (EMA), the US Food and Drug Administration (FDA) and other regulatory authorities (e.g. the Ministry of Agriculture, Livestock and Food Supply in Brazil) have strict safety requirements. In safety studies, products are usually tested at 1×, 3× and 5× their intended commercial doses. This is the case with recent studies on isoxazolines, which have demonstrated safety at the recommended dose [79, 100]. Indeed, the popularity of isoxazolines is largely due to their versatility of administration (topical or oral), the absence of reported resistance and a broad safety margin.

## Importance of veterinary leadership in One Health and how this relates to vector-borne diseases

The role of veterinarians in public health is reflected in the Veterinarian’s Oath adopted by the American Veterinary Medical Association’s House of Delegates in 1969 (amended in 1999 and 2010): “Being admitted to the profession of veterinary medicine, I solemnly swear to use my scientific knowledge and skills for the benefit of society through the protection of animal health and welfare, the prevention and relief of animal suffering, the conservation of animal resources, the promotion of public health, and the advancement of medical knowledge”.

**Fig. 5:**
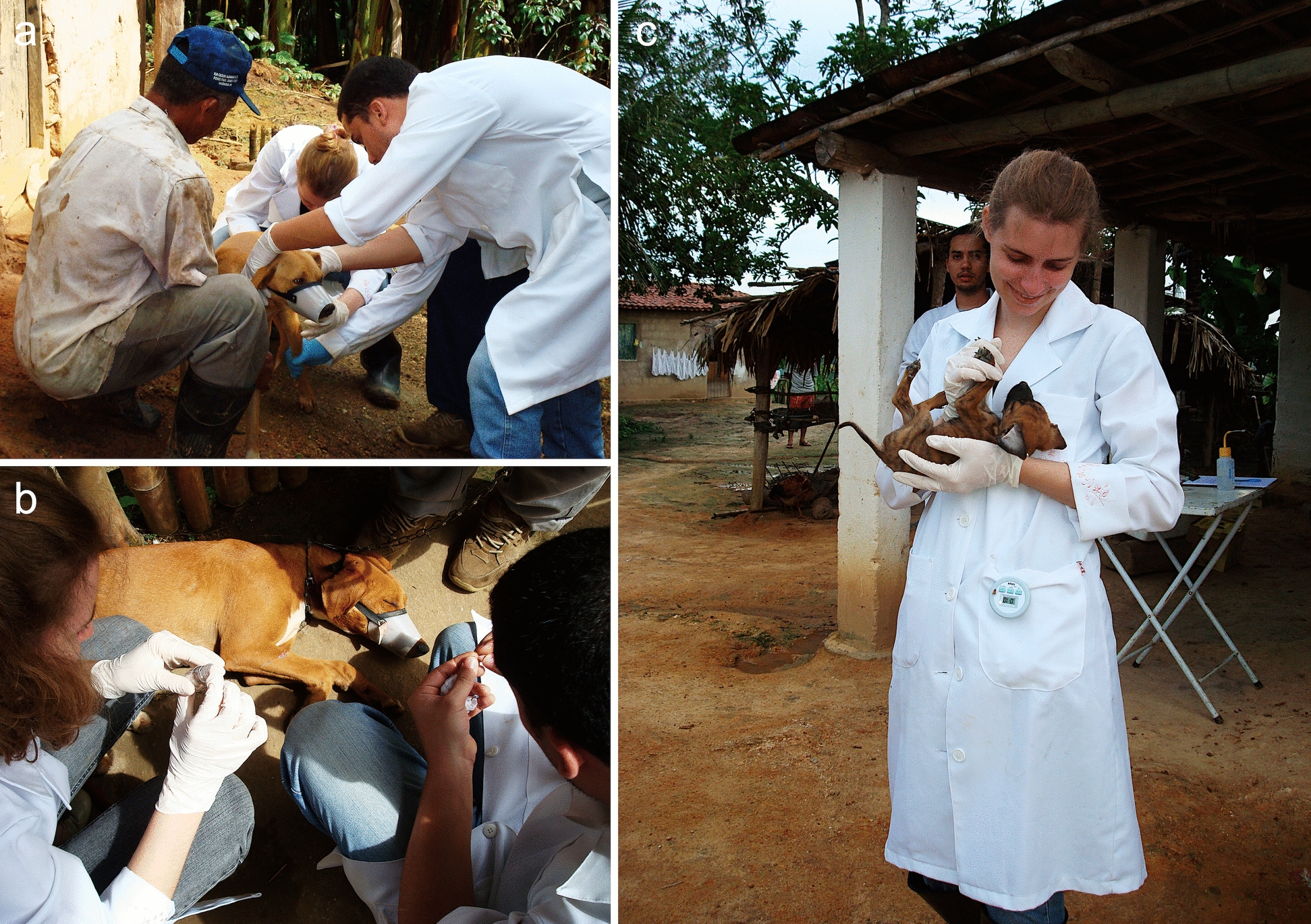
Veterinary researchers (**a**–**c**) studying vector-borne diseases in remote rural areas in northeastern Brazil (July 2008). Dog owners in these regions have limited access to healthcare and are at risk of zoonotic diseases, including leishmaniasis

It is also commonly said that while doctors heal individuals, veterinarians heal the world, underscoring their vital role in One Health, which extends beyond just caring for animals (Fig. 5). The crucial role of veterinarians in safeguarding human health was reinforced during the COVID-19 pandemic, through laboratory support for the diagnosis and molecular characterization of SARS-CoV-2 (severe acute respiratory syndrome coronavirus 2) and testing of human samples [101]. Yet the role of small-animal veterinary practitioners in protecting human health is often neglected. Veterinary practitioners primarily focus on animal health, frequently overlooking the zoonotic risks associated with pet ownership. This may have severe consequences in areas where clinically significant zoonotic diseases are present, including rabies, leishmaniasis, and echinococcosis.

The consultation moment is a unique opportunity for veterinary practitioners to reinforce their role as safeguards of animal and human health. A holistic view of the entire family and their environment is pivotal for developing individualized, effective prevention programmes to mitigate the risk of zoonotic diseases, including vector-borne diseases such as leishmaniasis and tick-borne rickettsiosis.

**Fig. 6:**
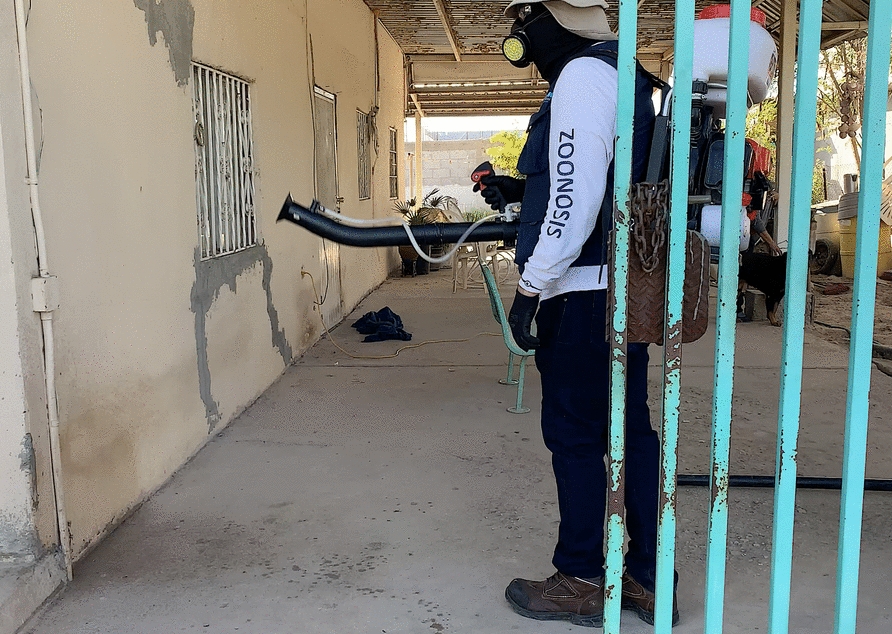
Public health agents applying pyrethroid-based products in residential areas in Mexicali (Mexico), where brown dog ticks have been identified. In this area, *Rhipicephalus linnaei* is the primary vector for Rocky Mountain spotted fever

Dogs act as sentinels for zoonotic vector-borne diseases [102]. When a zoonotic disease is detected in a dog, the veterinarian should, when notification is mandatory, officially report it to public health authorities and advise the pet owner to seek early medical attention if necessary. This proactive approach can lead to early diagnosis and treatment, which are vital for reducing the fatality rates from diseases such as Rocky Mountain spotted fever [17, 103]. The emergence of Rocky Mountain spotted fever in the southwestern USA and northern Mexico (Fig. 6) is a paradigmatic example that underscores the need for a collaborative One Health strategy to reduce case-fatality rates [102]. *Rickettsia rickettsii* infection may present with a flu-like illness in the initial phase, and delays in diagnosis and treatment may result in fatal outcomes [14]. In this perspective, it has been emphasized that veterinarians and physicians working in areas where *R. rickettsii* is endemic should inform about the need for early doxycycline treatment if symptoms arise [103]. Given that doxycycline is often prescribed for vector-borne diseases in dogs and humans, veterinarians must weigh the risks of antimicrobial resistance. This highlights the leading role of veterinarians in One Health.

Veterinarians should base their practices on evidence-based recommendations and consider the growing bond between dogs and humans. Many dogs are no longer seen merely as guard dogs, companions or pets; they are now regarded as family members. This shift even influences how we refer to dogs (from ‘it’ to ‘he’ or ‘she’) and to their owners (from non-parental terms like ‘owners’ to parental terms like ‘mom’ and ‘dad’). Such changes have completely transformed veterinarians’ view of their canine patients, prompting a shift in veterinary practice from a curative to a preventive approach. While the financial burden of treatment versus prevention can be directly measured and compared, the impact of a sick dog on the mental and physical health of their owners cannot be measured, especially in life-threatening conditions and fatal cases.

## Conclusions

Vector-borne infections are on the rise worldwide, particularly in tropical and subtropical regions. Some diseases are also expanding their geographical range in temperate areas, as climate change is making the environment more suitable for vectors that were previously absent there. While the risk of vector-borne pathogen transmission may vary seasonally in some places, vectors are often present year-round in most tropical and subtropical regions and are also increasing in temperate regions. In addition to their clinical significance for dogs, some vector-borne pathogens are zoonotic and cause life-threatening diseases in humans, including leishmaniasis and Rocky Mountain spotted fever. This underscores the importance of year-round prevention, as recommended by established international guidelines from leading veterinary parasitology organizations, to safeguard the health and well-being of dogs and their families, and to reduce global morbidity and mortality associated with these diseases.

## Data Availability

No datasets were generated or analysed during the current study.
